# Immune Evasion Strategies of Glioblastoma

**DOI:** 10.3389/fsurg.2016.00011

**Published:** 2016-03-02

**Authors:** Seyed-Mostafa Razavi, Karen E. Lee, Benjamin E. Jin, Parvir S. Aujla, Sharareh Gholamin, Gordon Li

**Affiliations:** ^1^Department of Neurosurgery, Stanford University School of Medicine, Stanford, CA, USA; ^2^Institute of Stem Cell Biology and Regenerative Medicine, Stanford University School of Medicine, Stanford, CA, USA

**Keywords:** glioblastoma, immune system, immunosuppression, immune evasion, cancer immunotherapy

## Abstract

Glioblastoma (GBM) is the most devastating brain tumor, with associated poor prognosis. Despite advances in surgery and chemoradiation, the survival of afflicted patients has not improved significantly in the past three decades. Immunotherapy has been heralded as a promising approach in treatment of various cancers; however, the immune privileged environment of the brain usually curbs the optimal expected response in central nervous system malignancies. In addition, GBM cells create an immunosuppressive microenvironment and employ various methods to escape immune surveillance. The purpose of this review is to highlight the strategies by which GBM cells evade the host immune system. Further understanding of these strategies and the biology of this tumor will pave the way for developing novel immunotherapeutic approaches for treatment of GBM.

## Introduction

**Figure 1:**
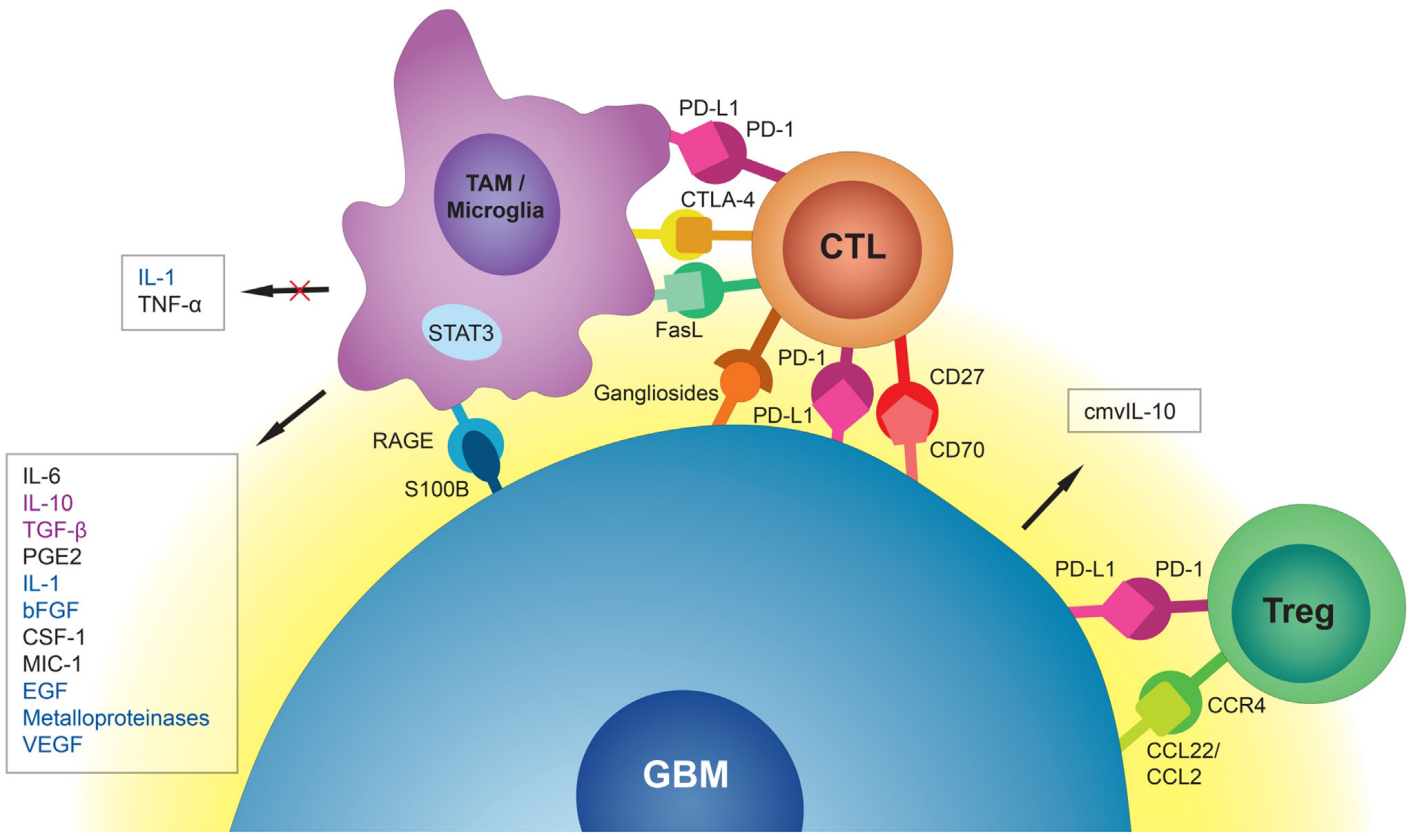
**Schematic representation of the GBM tumor cell interaction with surrounding immune environment**. Tumor-associated macrophages (TAMs) and microglia release immunosuppressive and pro-tumorigenic cytokines into the GBM microenvironment. They also induce cytotoxic T-cell (CTL) apoptosis via PD-L1, CTLA-4, and FasL. GBM cells, through interaction of S100B protein with receptor for advanced glycation end products (RAGE), inhibit production of immunostimulatory cytokines by TAMs and microglia. CMV-infected GBM cells secrete cmvIL-10 into their microenvironment with a range of immunosuppressive properties. Through interaction of CC chemokine ligand 22 (CCL22) and the weaker CC chemokine ligand 2 (CCL2) with CC chemokine receptor 4 (CCR4), GBM cells attract regulatory T-cells (Tregs) to the tumor site. Interaction of PD-L1 on GBM cells with PD-1 on Tregs promotes immunoregulatory functions of these cells. Immunosuppressive signals (black) could be distinguished from tumorigenic signals (blue) and signals that are both immunosuppressive and tumorigenic (purple).

One of the challenges scientists face in the treatment of glioblastoma (GBM) is suboptimal responses to immunotherapy (1, 2). GBM is the most common adult brain tumor and patients usually succumb to the disease in <2 years. Despite significant improvement in chemo- and radiotherapy approaches for treatment of GBM, the median survival of one and a half years has not seen a significant change in the past few years (3, 4). Stagnation in the treatment of GBM is attributable to different challenges in therapy and our poor understating of both tumor biology and interactions with its microenvironment. Due to infiltrative growth, local microscopic metastases, and sometimes presence of multiple lesions at the time of diagnosis (5), complete surgical excision of the tumor is practically impossible and there is a strong need for new and effective therapies. With the introduction of immunotherapy as a novel and promising approach to cancer treatment, new hopes are raised for the management of brain tumors. However, as far as GBM is concerned, immunotherapeutic strategies so far have not been able to prompt a great change in survival. This article aims to review the mechanisms employed by GBM cells to suppress and evade the body’s immune responses. The collection of different molecules and mechanisms discussed in this review are summarized in Table 1 and a schematic representation of the GBM tumor cell interaction with the surrounding immune environment can be found in Figure 1.

**Table 1:** **Summary of mechanisms employed by GBM to evade the immune system**.

Category^a^	Molecule/mechanism	Major source	Effect	Reference
Central nervous system	Blood–brain barrier	CNS anatomy	Prevents entry of immune cells	(6, 7)
	Glymphatic system	CNS anatomy	Carries immune cells and macromolecules	(8, 9)
	FasL/CD95L	Astrocytes	Induces T-cell apoptosis	(10, 11)
Microenvironment	IL-6	Microglia/TAMs	Suppresses immune effector cells	(12–14)
	IL-10	Microglia/TAMs	Enhances tumor growth, inhibits production of IFN-γ and TNF-α, down-regulates expression of MHC class II in monocytes, induces anergy in infiltrating T-cells	(15–20)
	TGF-β	Microglia/TAMs	Blocks T-cell activation and proliferation, inhibits IL-2 production, suppresses natural killer cell activity, promotes Treg activity, promotes tumor growth and invasion	(21–24)
	PGE2	Microglia/TAMs	Transforms DCs into regulatory phenotype	(25–29)
	IL-1	Microglia/TAMs	Promote tumorigenesis	(12–14)
	bFGF	Microglia/TAMs	Promote tumorigenesis	(12–14)
	CD70	GBM cells	Mediates T-cell apoptosis through interaction with CD27	(30, 31)
	Gangliosides	GBM cells	Induces T-cell apoptosis	(30, 31)
	FasL	Microglia/TAMs	Induces cytotoxic T-cell compromise and apoptosis	(32, 33)
	Hypoxia	Inappropriate vascularization/excessive oxygen consumption by GBM cells	Activation of Tregs through STAT3	(34–36)
Immune checkpoints	PD-L1	GBM cells, microglia/TAMs	Suppresses cytotoxic T-cell proliferation and function and activated Tregs by binding to PD-1	(37–43)
	CTLA-4	GBM cells	Modulates T-cell activation	(44, 45)
Regulatory T-cells	CCL22	GBM cells	Attracts Tregs to the tumor site by binding to CCR4	(46–48)
	CCL2	GBM cells	Attracts Tregs to the tumor site by weakly binding to CCR4	(46–48)
Tumor-associated macrophages	CSF-1	Microglia/TAMs	Polarizes TAMs toward M2 phenotype	(36, 49, 50)
	TGF-β1	Microglia/TAMs	Polarizes TAMs toward M2 phenotype	(36)
	MIC-1	Microglia/TAMs	Polarizes TAMs toward M2 phenotype	(36)
	IL-10	Microglia/TAMs	Polarizes TAMs toward M2 phenotype	(36)
	S100B	GBM cells	Inhibits production of pro-inflammatory cytokines by TAMs through STAT3 pathway	(51)
	EGF	Microglia/TAMs	Promotes tumor invasion and migration	(49, 52, 53)
	IL-6	Microglia/TAMs	Promotes tumor invasion and migration	(54)
	Metalloproteinases	Microglia/TAMs	Promotes tumor invasion and migration	(55)
	VEGF	Microglia/TAMs	Promotes tumor growth and vascularity	(56–58)
Human cytomegalovirus	cmvIL-10	Infected GBM cells	Impairs mononuclear cell proliferation, inhibits DC maturation and antigen presentation, suppresses inflammatory cytokine production, promotes TGF-β production, down-regulates MHC expression, prompts monocytes differentiation into M2 macrophages, upregulates PD-L1 on tumor cells	(59–63)

*^a^The section on antigen presentation is not given a separate category as the respective pieces of information are represented in other sections of the table*.

*CNS, central nervous system; IL, interleukin; IFN-γ, interferon-gamma; TNF-α, tumor necrosis factor-alpha; TGF-β, transforming growth factor beta; PGE2, prostaglandin E_2_; bFGF, basic fibroblast growth factor; DCs, dendritic cells; TAMs, tumor-associated macrophages; Tregs, regulatory T-cells; STAT3, signal transducer and activator of transcription 3; PD-L1, programed cell death ligand-a; PD-1, programed cell death protein-1; CTLA-4, cytotoxic T-lymphocyte antigen 4; CCL, CC chemokine ligand; CCR4, CC chemokine receptor 4; CSF-1, colony-stimulating factor-1; MIC-1, macrophage inhibitory cytokine-1; EGF, endothelial growth factor; VEGF, vascular endothelial growth factor*.

## Central Nervous System and the Immune System

The central nervous system (CNS), and more specifically the brain, has been historically presumed as the “immune privileged” organ of the body due to an intact blood–brain barrier (BBB). Absence of a usual lymphatic system and paucity of antigen-presenting cells (APCs) in brain tissue have also fueled this notion (6, 7). This assumption has been questioned in light of recent discoveries. The CNS possesses a functional “glymphatic system” located within the walls of dural sinuses and connected to the deep cervical lymph nodes capable of carrying immune cells and macromolecules (6, 8, 9). Immune cells can migrate into the brain parenchyma by chemotaxis, in which interferon-gamma (IFN-γ) and integrins play a major role (64, 65). Antigens can pass through walls of cerebral arteries and enter cervical lymph nodes through the Virchow–Robin perivascular spaces (66). By attaching to FcRn, a receptor found on a variety of body tissues, immunoglobulins are also able to cross the BBB via carrier-mediated transport (67, 68). APCs are present in many areas of the brain, including leptomeninges, ventricles, and perivascular spaces (69, 70). Via the rostral migratory stream, dendritic cells (DCs) can travel outside the brain and present antigens to T-cells located in the cervical lymph nodes (71). Peripheral immune cells can migrate to the CNS perivascular spaces but not into the brain parenchyma, thanks to the BBB. Tight junctions between foot processes of astrocytes form the physical BBB between perivascular spaces and parenchyma, while FasL/CD95L, expressed on these processes, induces apoptosis of T-cells that express the Fas receptor (10, 11). In disease states however, the integrity of the barrier is compromised, enabling immune cells to migrate past the BBB (72). During clinical trials for DC vaccines in patients with brain tumors, tumor-infiltrating lymphocytes have been observed in GBM samples (73, 74).

## Microenvironment

Functional immunosuppression in the GBM microenvironment is characterized by production of immunosuppressive cytokines, inhibition of T-cell proliferation and effector responses, activation of FoxP3+ regulatory T-cells (Tregs), and tissue hypoxia. Immunosuppressive cytokines, including interleukin (IL)-6, IL-10, transforming growth factor-beta (TGF-β), and prostaglandin E_2_ (PGE2), as well as tumor-promoting cytokines, IL-1, and basic fibroblast growth factor (bFGF), are present in the GBM microenvironment and dampen the antitumor immune response (12–14). TGF-β promotes immunosuppression in GBM by blocking T-cell activation and proliferation, inhibiting IL-2 production, suppressing natural killer cell activity, and promoting Tregs (21). In addition, TGF-β has been shown to promote tumor growth and invasion by supporting GBM stem cells and enhancing angiogenesis (22–24).

Generally known as an immunosuppressive cytokine, IL-10 is found at high levels in a variety of neoplasms (15, 16). This cytokine is secreted by various immune cells (mainly macrophages, but also helper and cytotoxic T-cells, DCs, B-cells, monocytes, and mast cells) as well as GBM cells (16, 17). IL-10 associated with GBM is shown to enhance tumor growth (18), inhibit production of IFN-γ and tumor necrosis factor-alpha (TNF-α) by the immune system, downregulate expression of MHC class II in monocytes, and, via the co-stimulatory CD28-CD80/86 pathway, induce anergy in infiltrating T-cells (19, 20).

PGE2 is known to promote regulatory immune response in cancers and stimulate tumor cell growth (25). Together with TGF-β, it transforms DCs into a regulatory phenotype that suppresses T-cell proliferation (26, 27). In the GBM microenvironment, however, the concentration of PGE2 is not found to be high enough to suppress T-cell functions on its own (28, 29).

The GBM microenvironment also mediates immunosuppression via mechanisms that increase T-cell propensity to apoptosis through a cooperative interaction between CD70 and gangliosides (30, 31). CD70, through interaction with CD27, a member of TNF receptor family proteins, mediates apoptosis in T-cells. Inhibition of gangliosides, components of the plasma membrane that modulate signal transduction events, causes GBM cells to be significantly less efficient at inducing T-cell apoptosis. It has been shown that blocking both CD70 and ganglioside function produces an additive effect on provoking T-cell apoptosis (31). Programed cell death protein-1 ligand (PD-L1, B7-H1, or CD274), a potent immunosuppressive molecule, is expressed on microglia. The expression of PD-L1 on microglial cells is increased when in proximity to GBM cells that can induce T-cell apoptosis (37–39). The role of PD-L1 as an immune checkpoint is discussed further in the respective section. Another immunoinhibitory molecule expressed on tumor-associated microglia is FasL, which can induce cytotoxic T-cell compromise and apoptosis. Inhibition of FasL has resulted in an increased number of immune cells within the tumor (32, 33).

Lack of oxygen in the GBM microenvironment is the result of morphologically inappropriate neovascularization, irregular blood flow, and excessive consumption of oxygen from rapidly proliferating tumor cells. Hypoxia is a strong stimulus for expression of genes involved in tumor cell growth and angiogenesis (34). Specifically, the hypoxic GBM microenvironment activates signal transducer and activator of transcription 3 (STAT3), an immunosuppressive pathway and potent regulator of anti-inflammatory responses, which triggers the synthesis of hypoxia-inducible factor-1α (HIF-1α) that subsequently induces activation of Tregs and production of vascular endothelial growth factor (VEGF) (34). Tregs are modulators of the immune response, and VEGF is known for its immunosuppressive effects. Additionally, the hypoxic microenvironment triggers CNS macrophages to transform into tumor-associated macrophages (TAMs), which then adopt immunosuppressive and tumor-supportive phenotypes (M2). This transformation, via the STAT3 pathway, induces TAMs to promote angiogenesis and tumor cell invasion (35). Additionally, it has been shown that TAMs are modulated by GBM cancer stem cells (gCSCs) through induction of an immunosuppressive phenotype via the STAT3 pathway (36). Furthermore, since HIF-1α promotes gCSCs, hypoxia likely causes a feed-forward mechanism in tumor-mediated immunosuppression.

## Antigen Presentation

Despite tremendous research, the mechanisms involved in developing tumor-sensitized immune effector cells are not well understood. Antigens from dead tumor cells are collected and processed by APCs and “cross-presented” on MHC class I to cytotoxic T-cells (75). Whether this antigen presentation for GBM occurs mainly in the brain or in the periphery is a subject of ongoing research (76). Microglia are the major myeloid immunocompetent cells of the brain, and scientists have elaborated their ability to present antigens to cytotoxic T-cells within the CNS (77, 78). However, the immunosuppressive microenvironment of GBM down-regulates MHC expression and compromises the antigen-presenting ability of microglia (79–83). GBM cells also stimulate secretion of IL-10 and inhibit production of TNF-α by microglia, further promoting suppression of the immune response (84). In fact, studies suggest that tumor-infiltrating DCs have a bigger part in GBM antigen presentation. In a 2008 study, Beauvillain et al. discovered that tumor-infiltrating DCs were more efficient than neonatal microglia in priming cytotoxic T-cells with exogenous antigens and could trigger higher levels of IL-2 and IFN-γ secretion by these cells (85). Presence of tumor-infiltrating DCs in the brain alongside microglia would prompt a better immune response in the CNS (77). Both glioma-associated antigen-pulsed and tumor-lysate-pulsed DCs have been successful in eliciting T-cell response in GBM patients (73, 74, 86). Wilms’ tumor 1 (WT1)-pulsed DC vaccine could improve neurological findings and shrink the tumor in a recent study (87). Nonetheless, tumor microenvironments would also blunt the action of tumor-infiltrating DCs and further investigation is needed to optimize this therapeutic technique (14, 20).

Macrophages are the major population of immune cells infiltrating solid tumors and GBM (88, 89). These cells are involved in antigen presentation, immune induction, cytotoxicity, removal of debris, regulation of inflammatory response, and thrombosis. Macrophages derived from monocyte precursors polarize into two distinct categories based on signals from the environment: M1, with a pro-inflammatory cytokine profile, and M2, with overall anti-inflammatory properties. Exposure to IFN-γ or bacterial lipopolysaccharide polarizes monocytes toward M1 macrophages. An alternate activation process happens by exposure to IL-4, resulting in the M2 category (90, 91). TAMs are believed to be of the latter population as they share many functions and surface proteins with M2 macrophages. While TAMs are known to be capable of cross-presenting tumor antigens to T-cells and prime antitumor immune response (92) due to limitations in histologic differentiation of TAMs from microglia, there is no definite answer to their importance in tumor antigen presentation in the brain (93, 94).

While mainly involved in humoral immune response, B-cells can also act as APCs and directly present antigens to T-cells via both MHC class I and II (95–97). Interaction of GBM cells with tumor-infiltrating B-cells has not been thoroughly investigated. Candolfi et al. studied the role of B-cells in a GBM murine model. After treatment of mice with intratumoral adenovector and immunostimulatory cytokines, B-cells were found to have remnants of tumor antigens in their cytoplasm and the ability to stimulate T-cell proliferation *in vitro* (98).

Tumor antigen presentation can also occur in peripheral lymph nodes. Activated T-cells have been found in the cervical lymph nodes of murine GBM models (99). Evidence exists that CNS antigens can move out of the CNS through perivascular spaces and be collected by resident DCs in cervical lymph nodes (100). Immunosuppressive cytokines secreted by GBM cells do not have a high enough systemic concentration to justify impairment of peripheral immune cell functions (101, 102). Engineered CTLs targeting IL-13 receptor 2 have shown promise in GBM *in vivo* models (103). Regardless of the underlying cause, vitiated cell-mediated immunity in GBM patients can compromise antigen presentation and T-cell activation even in the peripheral lymphatic tissue, adding to the challenges of immunotherapeutic efforts.

## Immune Checkpoints

Immune checkpoint molecules, a group of co-stimulatory and co-inhibitory pathways that limit the function of immune system, have recently been targets for extensive research. By inhibition of immune checkpoints, researchers were able to reverse immunoresistance of cancer cells and activate the immune cells against tumors (104).

A major immune checkpoint molecule implicated in GBM immune evasion is PD-L1. Modulated by the PI(3)K–Akt–mTOR pathway (38), PD-L1 suppresses proliferation and function of cytotoxic T-cells and promotes Tregs activity by binding to programed cell death-1 (PD-1) (40). Expression of PD-L1 on tumor cells and T-cells is correlated with tumor grade (41) and poor survival of GBM patients (42). Microglia and TAMs are also known to express PD-L1 on their surface and at the same time promote PD-L1 expression on GBM cells (37, 43, 105). Collectively, these findings have made this immune checkpoint a prime target for GBM immunotherapy. Pre-clinical studies have been promising (106, 107) with plans for clinical trials on GBM patients currently under way.

Another immune checkpoint molecule, cytotoxic T-lymphocyte antigen 4 (CTLA-4) expressed on activated T-cells and Tregs could play a role in GBM immune evasion. Targeting CTLA-4 in GBM models might be able to enhance antitumor activity by T-cells (44, 45). Immune checkpoint inhibitors as targeted cancer therapeutics have shown promise in recent years with researchers trying to find new checkpoints as immunotherapeutic targets.

## Regulatory T-Cells

Tregs, a small population of CD4+ T-cells that specifically express FoxP3 transcription factor, are a group of circulating lymphocytes with suppressive effects on various immune cells (108, 109). Other markers that help distinguish Treg subpopulations are CD25 (high-affinity IL-2 receptor), CTLA-4, and glucocorticoid-induced tumor necrosis factor receptor (110). Tregs can be divided into two major subpopulations based on their origin. Thymus-derived Tregs, developed from naïve CD4+ cells after antigen presentation in the thymus, express high levels of FoxP3. By contrast, under IL-10 and TGF-β signaling in the periphery, conventional CD4+ T-cells differentiate into peripherally induced Tregs with negligible FoxP3 expression (109). Tregs are commonly known to regulate immune response against tumor cells and to shift the tumor cytokine milieu toward immunosuppression. The presence of Tregs in GBM patients was described years ago (111), but their intricate function and interaction with other cells is a matter of ongoing investigation. A higher population of Tregs is demonstrated in GBM patients, reported to comprise up to 25% of tumor-infiltrating lymphocytes, and their abundance is associated with poor prognosis (112–114). Studies have revealed that glioma-associated Tregs are mostly of thymic origin rather than tumor-derived (115), suggesting that the abundance of Tregs in GBM is a result of chemotactic attraction of the thymus-derived subpopulation rather than local differentiation in the tumor (116). The CC chemokine ligand 22 (CCL22) and the weaker CC chemokine ligand 2 (CCL2) are among the first molecules revealed to attract Tregs to the tumor site by binding to CC chemokine receptor 4 (CCR4) (46, 47). Further studies revealed that blocking this receptor cannot completely abrogate Treg infiltration into GBM tumor mass, suggesting involvement of other secretory molecules in Treg chemoattraction (48). Peripherally derived Tregs are not believed to be the major population of Tregs in GBM, but presence of IL-10 and TGF-β at high levels in the GBM microenvironment suggests the possibly noticeable role of these cells in immune evasion of the tumor (14, 109). Further studies are needed to reveal the holistic picture of Tregs recruitment mechanisms into GBM.

## Tumor-Associated Macrophages

Involvement of macrophages in GBM progression is a question to be further investigated. Recent studies provide significant evidence in contextual response of macrophages in tumor progression, highly modulated by the tumor microenvironment and tumor response to conventional treatments. Distinguishing TAMs from microglia in the brain is still a challenge for researchers. While TAMs are found to have a high expression of CD11b and CD45 compared to microglia, which have high expression of CD11b but low expression of CD45, there is still disagreement over a universally accepted histological marker that distinguishes the two cell types (117, 118).

Tumor-associated macrophages are usually linked to accelerated disease progression and poor outcome in cancer patients (119–121). Recently, several approaches have been investigated to abrogate tumor progression through ablating TAMs. Modulating the routes involved in macrophage polarization has provided insight into the regulatory effect of these cells in the GBM microenvironment (122).

Innate immunosuppressive properties of gliomas are derived from the regulatory cross-talk between M2 phenotype macrophages and tumor cells (93). Macrophages and microglia as dominant populations of tumor-infiltrating immune cells are, to a great extent, regulated by glioma initiating cells. Upon chemoattraction into the tumor environment (47, 49, 123, 124) with a high concentration of colony-stimulating factor-1 (CSF-1), TGF-β1, macrophage inhibitory cytokine-1 (MIC-1), and IL-10, TAMs are polarized toward the M2 phenotype, subsequently inhibiting their phagocytic ability and enhancing their capacity to inhibit cytotoxic T-cell proliferation and increase the effect of Tregs (36). Inhibiting the CSF-1 receptor can shift the polarization of TAMs away from M2, hinder their tumor-promoting functions, and increase survival of the GBM-bearing mice (50). Another protein recently found on GBM cells to induce innate immune suppression is S100B. Through interaction of S100B with receptor for advanced glycation end products (RAGE) on macrophages, GBM cells induce the STAT3 pathway in TAMs and inhibit the production of IL-1β, TNF-α, and other pro-inflammatory cytokines by these cells (51).

Tumor-associated macrophages and microglia can also play a role in GBM growth, invasion, and angiogenesis. Endothelial growth factor (EGF), CSF-1, TGF-β1, IL-6, and metalloproteinases originating from TAMs and microglia are instrumental for glioma invasion and migration (49, 52, 54, 55, 125). Inhibition of the EGF receptor (EGFR) on GBM cells has been associated with antiangiogenic and proapoptotic effects on the tumor (53). Inhibition of VEGF signaling in TAMs and microglia leads to decreased GBM growth and vascularity (56), but addition of anti-VEGF-A antibody to standard treatment has not improved patient survival (57, 58). Other populations of cells from myeloid lineage have been found in gliomas, including tumor-associated neutrophils, angiogenic monocytes, and immunosuppressive myelomonocytic cells, the importance of which is yet to be elucidated (126).

## Human Cytomegalovirus Infection

Human cytomegalovirus (HCMV) is a β-herpesvirus implicated in GBM pathogenesis. Different studies have found HCMV genome in most tested GBM samples with no trace of the virus in surrounding brain tissue (59, 60). The role of HCMV in GBM development and pathogenesis is not yet clarified. What is clear though is that HCMV infection could play a role in immunosuppression in the context of GBM microenvironment.

Human cytomegalovirus genome encodes an IL-10 homolog (cmvIL-10) – a product of UL111A gene – that could impair mononuclear cell proliferation, inhibit DC maturation and antigen presentation, suppress inflammatory cytokine production, and down-regulate MHC expression (61, 62). Moreover, it has been demonstrated that cmvIL-10 prompts monocytes to differentiate into M2 macrophages and up-regulates the immunoinhibitory PD-L1 protein on GBM cells. Additionally, monocytes treated with cmvIL-10 produce TGF-β, augmenting the immunosuppressive microenvironment (63).

## Summary and Future Prospects

The interaction of GBM with the immune system is intricate at every level. Any of the various mechanisms employed by this tumor to evade and suppress the immune response could be targeted with immunotherapy. To date, trials of immunotherapeutic modalities for GBM have not been as successful as promised. As different mechanisms of GBM immune resistance are revealed, scientists could have a better understanding of the pitfalls in GBM immunotherapy. GBM strategies for immune evasion are diverse and the key to successful immunotherapeutic treatment seems to be in targeting several pathways at the same time.

## Author Contributions

SMR organized and wrote major parts of the article. KL helped with writing the article. BJ helped with writing the article. PA helped with designing the figure. SG helped with writing the article.

## Conflict of Interest Statement

The authors declare that the research was conducted in the absence of any commercial or financial relationships that could be construed as a potential conflict of interest.
